# Termination-Dependent Surface Fermiology in the Intercalated Altermagnet CoNb_4_Se_8_

**DOI:** 10.1021/acs.nanolett.6c01819

**Published:** 2026-10-19

**Authors:** Pietro Maria Forcella, Alessandro De Vita, Matthew D. Watson, Sammar Tayyab, Chiara Bigi, Iulia Cojocariu, Marcin Szpytma, Matteo Jugovac, Andrea Locatelli, Tevfik Onur Menteş, My Bang Petersen, Paolo Moras, Polina M. Sheverdyaeva, Christopher Candelora, Ilija Zeljkovic, Federico Motti, Jill A. Miwa, Vincent Polewczyk, Matthieu Jamet, Matteo Calandra, Younghun Hwang, Davide Romanin, Federico Mazzola

**Affiliations:** † 129826 Université Paris-Saclay, CNRS, Centre de Nanosciences et de Nanotechnologies 91120 Palaiseau, Paris, France; ‡ 28259 Fritz Haber Institut der Max Planck Gesellschaft, Faradayweg 4−6, 14195 Berlin, Germany; § 120796 Diamond Light Source Ltd, Harwell Science and Innovation Campus, Didcot OX110DE, United Kingdom; ∥ Dipartimento di Fisica, 9311 Sapienza Università di Roma, P.le Aldo Moro 2, 00185 Rome, Italy; ⊥ 55536 Synchrotron SOLEIL, L’Orme des Merisiers, Départementale 128, F-91190 Saint-Aubin, France; # Elettra - Sincrotrone Trieste S.C.p.A., Strada Statale 14, km 163.5, 34149 Trieste, Italy; 7 Dipartimento di Fisica, Università degli Studi di Trieste, 34127 Trieste, Italy; 8 Faculty of Physics and Applied Computer Science, 49811 AGH University of Krakow, Władysława Reymonta 19, 30-059 Krakow, Poland; 9 567504 CNR-Istituto di Struttura della Materia (CNR-ISM), Strada Statale 14, km 163.5, 34149 Trieste, Italy; 10 Department of Physics and Astronomy, Interdisciplinary Nanoscience Center, 1006 Aarhus University, 8000 Aarhus C, Denmark; 11 Department of Physics, 6019 Boston College, Chestnut Hill, Massachusetts 02467, United States; 12 CNR-Istituto Officina dei Materiali (IOM), 9315 Unità di Trieste, Strada Statale 14, km 163.5, 34149 Basovizza (TS), Italy; 13 27048 Université Paris-Saclay, UVSQ, CNRS, GEMaC, 45 Av. des États Unis, 78000 Versailles, France; 14 70610 Univ. Grenoble Alpes, CEA, CNRS, IRIG-SPINTEC, 17 Av. des Martyrs, 38054 Grenoble, France; 15 Department of Physics, 19034 University of Trento, Via Sommarive 14, Povo 38123, Italy; 16 Center for Artificial Low-Dimensional Electronic Systems, Institute for Basic Science (IBS), Pohang 37673, Republic of Korea; 17 Electricity & Electronics and Semiconductor Engineering, 65683 Ulsan College, Ulsan 44610, Republic of Korea; 18 Department of Physics and Astronomy ‘Galileo Galilei’, 9308 University of Padova, Via Francesco Marzolo, 8, 35121 Padova, Italy; 19 Institut für Physik und Astronomie, Technische Universität Berlin, Straße des 17 Juni 135, 10623 Berlin, Germany

**Keywords:** Altermagnetism, CoNb4Se8, Intercalated van der Waals, ARPES, DFT

## Abstract

Altermagnets have recently gained attention for hosting symmetry-enforced spin-split bands despite zero net magnetization. Angle-resolved photoelectron spectroscopy (ARPES) offers direct access to their electronic structure. However, the intrinsic surface sensitivity of this technique complicates the identification of the observed features: Surface states can be misinterpreted as the spin-split counterpart of bulk bands. This issue becomes particularly relevant for layered intercalated systems, where micrometer-sized domains with distinct terminations often coexist on a cleaved surface. We discuss this scenario for CoNb_4_Se_8_, one of the first established layered altermagnets. Supported by density functional theory calculations, we employ micro-ARPES to probe individual domains and reveal two contrasting surface terminations. We identify Co- and Se-terminated domains with characteristic surface states arising from bulk-surface connectivity of the material. Our work shows that termination-dependent surface contributions are essential for the reliable spectroscopic identification of altermagnetism and for assessing its presence across the family of layered materials.

## Graphical abstract

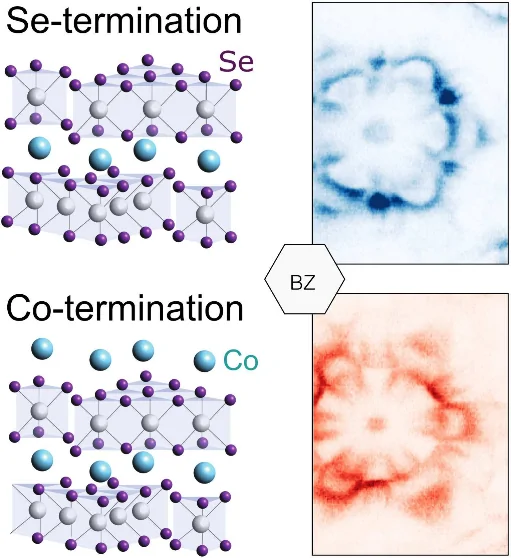

Altermagnetism has recently emerged as a fundamental class of magnetism in which specific crystalline symmetries enforce robust spin-split bulk electronic bands despite zero net magnetization.^1–4^ This effect was first proposed in theoretical works predicting nonrelativistic momentum-dependent spin polarization in antiferromagnets,^1,5,6^ and later observed experimentally in material candidates such as MnTe, RuO_2_, and CrSb.^7–10^ Angle-Resolved Photoelectron Spectroscopy (ARPES) is a powerful tool for uncovering such unconventional spin splittings in real materials.^11^ However, the interpretation of ARPES measurements is often complicated by surface-related contributions, as exemplified by the case of RuO_2_.^10,12,13^

Co-intercalated NbSe_2_ compounds have attracted interest for exhibiting symmetry conditions compatible with an altermagnetic band structure. CoNb_4_Se_8_, composed of Co atoms intercalated between NbSe_2_ layers, hosts a collinear antiferromagnetic ground state and has been proposed as one of the first realizations of altermagnetism in layered materials.^14–16^ Electronic-structure calculations predict sizable spin splitting and a highly anisotropic spin texture, and spectroscopic measurements report bulk-like features showing nonzero spin polarization at multiple *k*-points, consistent with an altermagnetic electronic structure.^15^ While previous studies focused on bulk properties, surface effects are expected to play a crucial role in altermagnets. The symmetry-enforced, momentum-dependent spin splitting of the bulk can be lost at the surface;^17^ specifically, a single-layer projection of the electronic structure of CoNb_4_Se_8_ along the *c* axis ([001] direction) implies *k*_*z*_ = 0 and thus a nodal plane, enforcing spin-degenerate states in the whole Brillouin zone. In addition, surface states can obscure bulk bands.^17–19^ Despite the possibility of themselves mediating exotic phases,^20,21^ in this context surface states challenge the clear identification of bulk altermagnetic features in the material.^22^

Moreover, some experimental challenges require serious attention. For example, cleaving the intercalated CoNb_4_Se_8_ can expose inequivalent atomic planes, i.e., chalcogen- versus intercalant-terminated surfaces, each characterized by a distinct surface potential and hosting its own set of surface bands. When these termination-dependent states disperse near the bulk altermagnetic bands, they can modify the apparent bulk-surface connectivity and be misinterpreted as additional spin-split branches, thereby distorting the interpretation of the inferred electronic structure. The importance of this effect is exemplified by the magnetic Weyl kagome metal Co_3_Sn_2_S_2_, where micro-ARPES and first-principles calculations have shown that different surface terminations host distinct topological surface states and even alter the connectivity of Weyl points while leaving the underlying bulk nodes intact.^18^ Standard ARPES is also intrinsically limited in resolving termination-dependent electronic structures in layered materials: typical termination domains extend over only a few tens of microns,^18,23^ thus the photon footprint inevitably averages over multiple surface regions, and spectral broadening masks the distinct dispersions associated with individual terminations. Such averaging can wash out termination-specific states or merge them into apparent bulk features, making it challenging to disentangle genuine altermagnetic band splittings from surface-derived replicas. These limitations are particularly severe for CoNb_4_Se_8_, whose structure naturally permits both chalcogen and intercalant terminated surfaces (see Figure 1a).^15,16^ As a result, conventional spectroscopies cannot determine whether observed spectral features originate from the intrinsic bulk altermagnetic band structure or instead from surface-localized states associated with one or more terminations. Despite these precedents, the termination-dependent surface electronic structure of layered altermagnets such as CoNb_4_Se_8_ has not yet been characterized.

**Figure 1:**
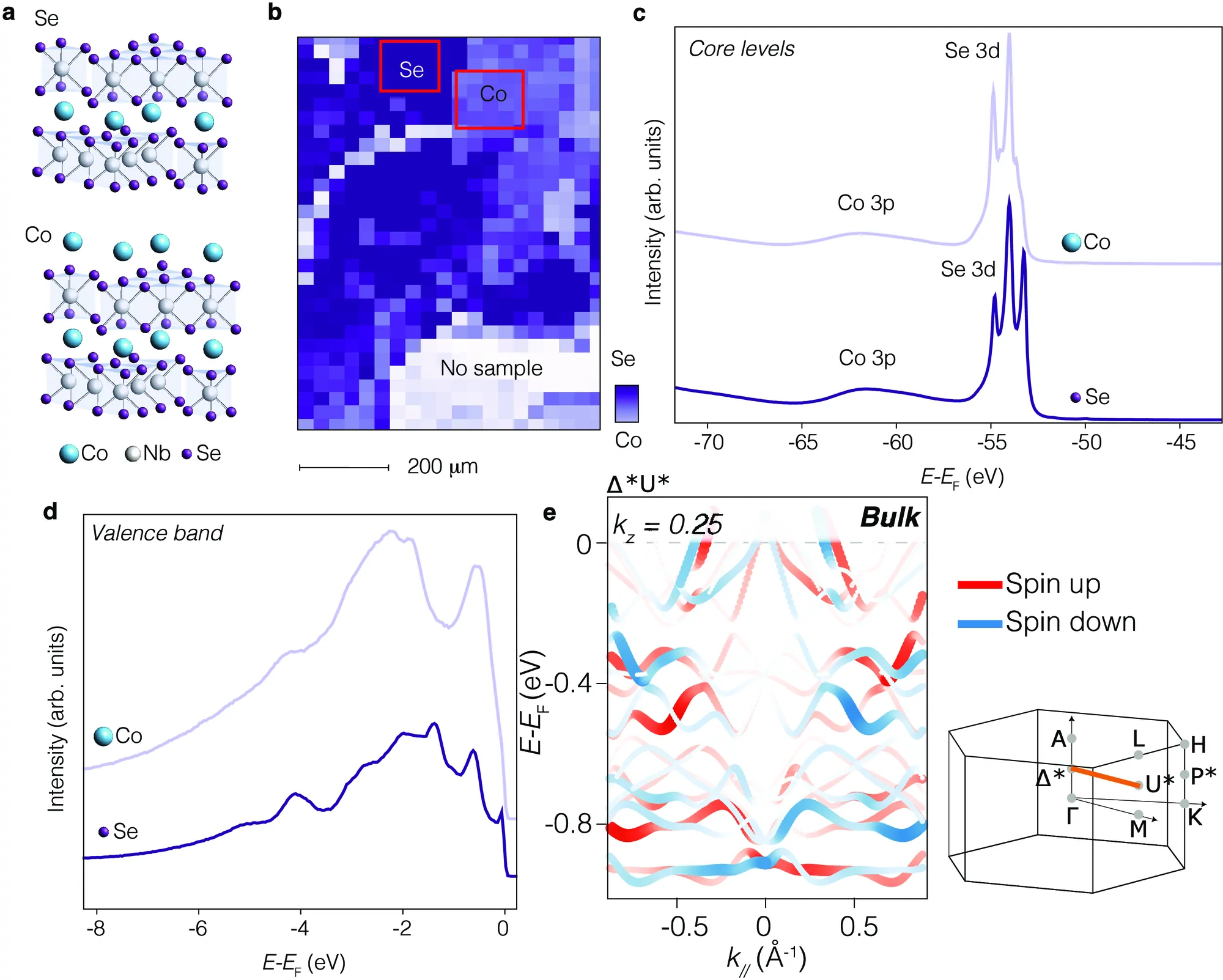
**a.** Schematics of the crystal structure and the possible surface terminations obtained after cleaving the sample. **b.** Micro-ARPES spatial map obtained by raster-scanning the beam and integrating, at each pixel, the valence-band intensity over the termination-sensitive window *E* ∈ [43.0, 43.13] eV and *k* ∈ [−1.85, – 0.85] Å^–1^. This window was chosen from the single-domain spectra and slab-DFT fingerprints and is used as a spatial guide only; the final termination assignment is made spot-by-spot from the agreement between the core-level line shape and the near-*E*_*F*_ band fingerprint, without applying an automated intensity-ratio threshold. Here for simplicity we employ deep-purple for Se, light purple for Co, and white for absence of signal. **c.** The distinct surface character is supported also by microresolved core-level X-ray photoelectron spectroscopy on the same spots acquired at 120 eV photon energy, showing features reconcilable with Co and Se surface spectra and in accordance with previous works. **d.** Valence band photoemission spectroscopy signal for the two terminations, acquired at 120 eV photon energy. As detailed in the main text, the differences are reflected by an overall surface doping due to the presence of a different termination and the presence of surface states. **e.** For completeness, we show consistently with ref 15., the calculated bulk electronic structure along the direction indicated in the Brillouin zone, featuring a spin-pattern consistent with altermagnetism.

To overcome these challenges, we employed microresolved experimental techniques for the characterization of the surface electronic properties of CoNb_4_Se_8_. Micro-ARPES allowed us to probe selectively two single-surface terminations, i.e., Co and Se. By reducing the probing area well below the characteristic domain size, our measurements granted direct access to the termination-dependent electronic structure of CoNb_4_Se_8_ without artifacts arising from spatial averaging. The excellent correspondence of experimental data with first-principle Density Functional Theory (DFT) calculations fully proves the attribution to the Co- and Se-terminated areas. Our results provide a comprehensive overview of the surface contribution to the Fermiology in CoNb_4_Se_8_, while also establishing a methodological precedent for robust distinction of bulk altermagnetic features from surface-derived bands.

Single crystals of CoNb_4_Se_8_ were grown by chemical vapor transport following procedures described in our previous works on Co-intercalated NbSe_2_,^15,16^ where we demonstrated that controlled Co intercalation between NbSe_2_ layers leads to a doubling of the crystallographic unit cell, producing a robust 2 × 2 modulation visible across a broad temperature range. Some of the photoemission-resolved data sets analyzed here were acquired as part of the experimental campaign reported in ref 15. In some cases, measurements were performed on the same samples, on different cleaves. Full synthesis details and characterization procedures for the present crystals are reported in the Methods section of ref 15., and in the Supporting Information. Micro-ARPES measurements were performed at the I05-Nano branch of Diamond Light Source (DLS), providing spectra with a lateral spatial resolution better than 4 μm. The full description of the DFT calculations methods^24–30^ can be found in the Supporting Information.^31^

We stress that the termination assignment used here does not contradict the real-space STM periodicities reported previously for CoNb_4_Se_8_.^15,16^ The 2*a*_0_ × 2*a*_0_ modulation is a structural consequence of the ordered Co intercalation, which doubles the in-plane unit cell of the parent 2*H*-NbSe_2_, and is therefore common to both cleavage terminations rather than being a unique fingerprint of only one surface. Its STM contrast and Fourier-transform amplitude can differ strongly between terminations because STM probes either a projected modulation of the outer Se lattice, with Fourier components at half the Se Bragg wavevector, or the more direct residual ordered Co ad-lattice left on the surface after cleaving. Within this picture, additional Fourier features reported previously reflect termination-dependent tunnelling matrix elements, surface sensitivity, and local disorder, rather than a structural inconsistency with the present assignment.

We first establish the existence of two distinct surface terminations in CoNb_4_Se_8_. This is directly visualized using micro-ARPES, as shown in Figure 1b, where photoemission intensity maps reflect the chemical heterogeneity present on the surface. At each spatial position, Se 3*d* and Co 3*p* core-levels are monitored, enabling the identification of distinct surface features reconcilable with surface-localized Co and Se, as illustrated in Figure 1c for representative regions. From core level analysis (See Supporting Information Figure S1 for fits and quantitative details), the investigated areas show a variation in the relative Co vs Se signal, consistent with distinct terminations. Se-terminated regions exhibit additional peaks due to surface core levels shift, while in the Co-terminated regions such replicas are significantly suppressed in intensity. The two terminations also exhibit markedly different valence-band spectra: angle-integrated measurements (Figure 1d) show distinct lineshapes consistent with different surface potentials, while both terminations retain metallic character with bands crossing the Fermi level. The density of states near *E*_*F*_ has mixed atomic character, with termination-dependent Nb, Se, and Co contributions; the Se and Co components are larger for the corresponding termination, as shown by the atom-projected PDOS in the Supporting Information (Figure S5).

However, a quantification by means of (angle-integrated) XPS carries a large uncertainty, associated both with the inelastic mean free path and with the possibility of additional Se features tailing into the Co region, the relevant peaks being very close to one another; XPS therefore is able to provide only circumstantial evidence and is intended to support, rather than establish, the two-termination picture. For this reason, to elucidate the surface-state Fermiology and hereby provide decisive evidence for the attribution of surface terminations, we acquired both Fermi surface maps and high-resolution energy-momentum maps using micro-ARPES, focusing exclusively on well-ordered regions exposing the features sketched in Figure 1. We therefore label these measurements accordingly as “Se-term” and “Co-term”. The Fermi surfaces (shown in Figure 2a in blue and Figure 2c in red, respectively) both exhibit flower-like patterns, yet they differ markedly already at first inspection; for example, they feature different shapes in the Fermi surface. Notably, these data sets were acquired at the same photon energy, polarization, and experimental conditions, ensuring a direct and reliable comparison. For both situations, the Fermi surfaces do not share electronic structures similar to the undoped parent compound NbSe_2_,^32,33^ but the Co intercalation drastically modifies the Fermiology.

**Figure 2:**
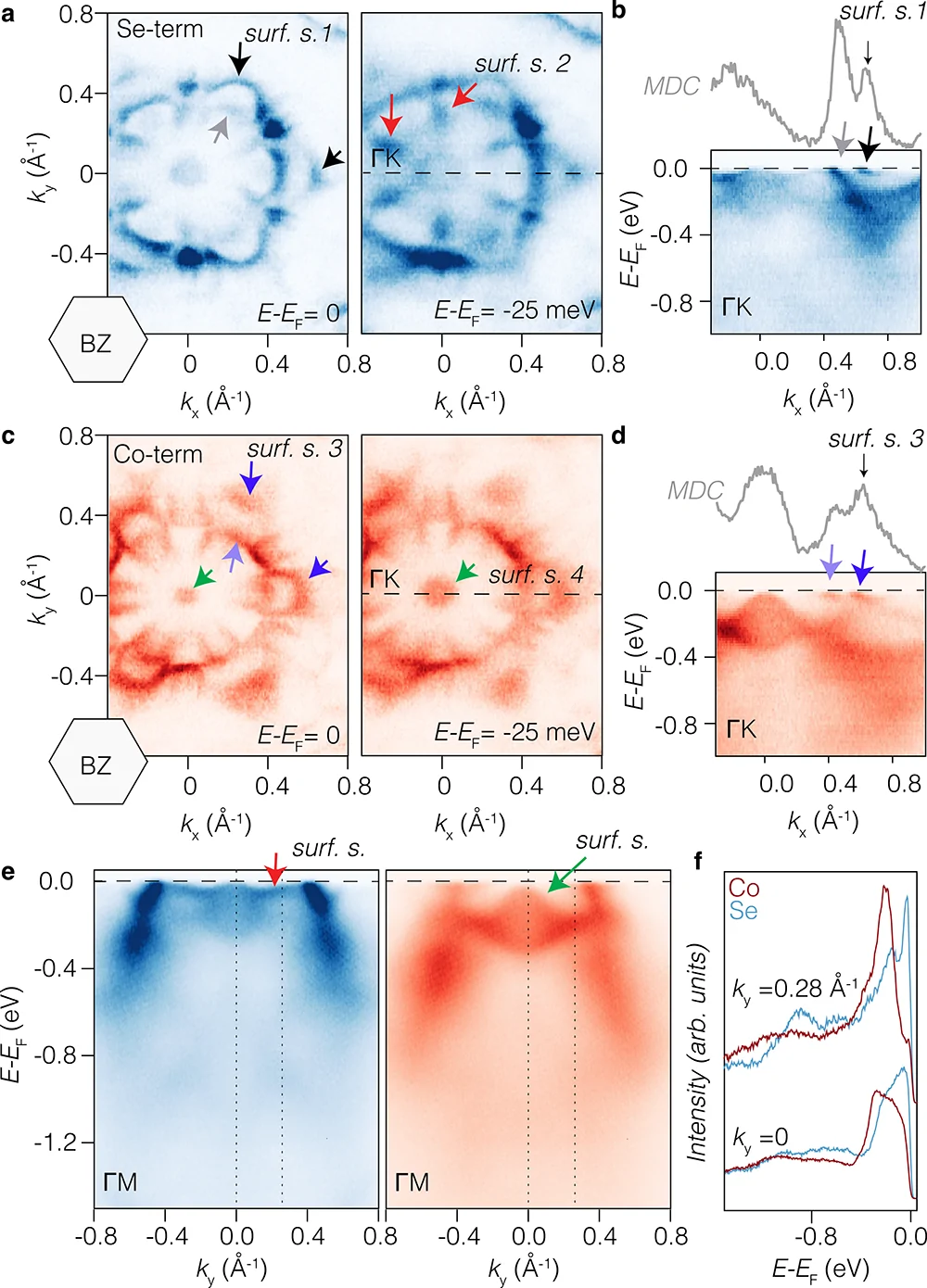
Fermi surface (*left*) and constant energy contour at *E* – *E*_*F*_ = – 25 meV (*right*) for the **a.** Se and **c.** Co terminations. The intensity shows a 3-fold symmetric pattern with elongated petals. The arrows indicate termination-dependent surface-derived features or surface resonances; same colors track the same features across the spectra. Such states are prominent especially along the Γ-K directions, shown in **b.** for Co and **d.** for Se. Along with the ARPES spectra, momentum distribution curves (MDCs) at the Fermi level are reported. **e.** Spectra along Γ-M for both terminations are compared. While there are near-Fermi level features visible in the Se-termination, these are absent in the Co one. An overall 40 meV shift of the Fermi energy is also observed consistent with electron doping provided by the presence of Co at the top surface. However, even by shifting the Fermi level down by 60 meV for the Co-termination spectra, the resulting energy distribution curves extracted at different momenta (dotted vertical lines in **e.**), shown in **f.**, still look significantly different (see also the comparison with constant energy maps in Supporting Information, Figure S2).

The constant energy surface pattern becomes even more evident in constant-energy maps extracted below the Fermi level, as illustrated by the data at 25 meV binding energy (BE) in Figure 2a-b. For constant energy maps at additional binding energies, see also the data presented in Supporting Information Figure S2. The termination-dependent differences are further clarified by analyzing momentum-resolved photoemission spectra along the high-symmetry directions Γ̅ – *K̅* and Γ̅ – *M̅*, as shown in Figure 2b-e. For both terminations, sharp dispersive features are observed along Γ̅ – *K̅* and cross the Fermi level. These states are clearly separated from the broader valence-band spectral weight and are labeled “surf. s. 1–3” in the figure, additionally they accounts for the arc-like intensity observed in the corresponding Fermi-surface maps (Figure 2a,c).

Additional termination-dependent features become evident in constant-energy maps acquired at finite binding energies. At *E* – *E*_*F*_ = – 25 meV, the Se-terminated surface exhibits elongated intensity segments arranged with an overall 6-fold symmetry, whereas no analogous pattern is observed for the Co-terminated surface. Consistently, the dispersions measured at *E* – *E*_*F*_ = – 25 meV on the Se termination show minimally dispersing bands along Γ̅ – *M̅* in a “cartwheel spoke” fashion (“surf. s. 2”) around a central “bore”-like pocket at Γ̅. These states are absent for the Co-terminated surface (Figure 2e). The contrast between the two terminations is further emphasized by energy distribution curves (EDCs) extracted at selected momenta (Figure 2f), which reveal clear differences in both peak positions and spectral weight.

We point out that the differences between the band texture of the two terminations cannot be explained solely by local doping variations across the surface. This is demonstrated by analyzing a series of constant-energy maps (Supporting Information, Figure S2). The central Γ̅-centered pocket is not used here to assign the termination-dependent states, but only as a common registration feature to test the rigid-shift hypothesis; its precise bulk, surface, or surface-resonance character is not essential to our conclusions. Matching its area requires a finite energy shift between the two terminations, confirming a local chemical-potential offset; however, once this shift is applied, the remaining petal- and spindle-like features still do not overlap in shape, connectivity, or spectral weight. The two Fermiologies therefore cannot be explained by a rigid shift alone, and instead reflect distinct termination-dependent surface or surface-resonance contributions.

The experimental observations described above find direct correspondence in density functional theory (DFT) calculations (Figure 3), which explicitly account for the two possible surface terminations. By modeling slab geometries with Se- and Co-terminated surfaces, we compute both the Fermi surfaces and the momentum-resolved photoemission spectra. Details of the calculations are reported in the Supporting Information, along with bulk-projected and slab spectra plotted separately for both terminations and along both Γ-M and Γ-K (Figure S3). In the assessment of the DFT-calculated spectra, we classify a feature as an isolated termination-derived surface state only when two criteria are satisfied: (i) it lies in an energy–momentum region where no bulk band is present in the bulk projection, i.e., outside the gray bulk continuum, and (ii) its dispersion and spectral weight depend on the termination, appearing for one termination while being absent, or different, for the other. When a termination-dependent feature overlaps the projected bulk continuum, we do not classify it as an isolated surface state but describe it as a surface resonance. This distinction is supported by the atom- and layer-projected DOS shown in the Supporting Information (Figures S5 and S6). The calculations accurately reproduce the key experimental features, including the petal-like Fermi-surface contours (black arrows), the spindle-shaped states (red arrows for Se and gray arrows for Co), and the pronounced termination-dependent differences below the Fermi level (Figure 3a-b). In particular, surface-derived bands appear selectively for one termination while remaining absent for the other, consistent with the termination-specific states identified by micro-ARPES. The same petal- and spindle-like features are also evident in the calculated energy-momentum dispersions of Figure 3c,d along the Γ-K direction, where well-defined surface-derived bands approach and intersect the Fermi level, in agreement with the arc-like intensity observed experimentally. By contrast, along Γ-M no states with clear surface character cross *E*_*F*_ at similar *k*-values, indicating that this direction does not contribute prominently to the petal surface-state manifold at the Fermi surface. Nevertheless, a closer inspection of the dispersions at lower *k*-values reveals a rich termination-dependent structure also along Γ-M. In particular, for the Se-terminated surface two weakly dispersive bands appear just below *E*_*F*_, forming locally flat features encompassed by the bulk states, whereas the Co-terminated surface exhibits a distinct diamond-shaped state shifted to lower binding energy, as highlighted in Figure 3c-d. In particular we note that, for both terminations, along Γ-M surface-derived contributions disperse closely alongside the bulk bands. This proximity identifies these overlapping features as surface resonances and shows how surface-derived spectral weight can obscure or mimic bulk altermagnetic features when contributions from different terminations are spatially averaged. On the other hand, micro-ARPES disentangles the tiny termination-induced spectral weight shifts, and can therefore univocally select one termination where bulk altermagnetic features are not mixed with multiple surface Se- and Co-induced contributions.

**Figure 3:**
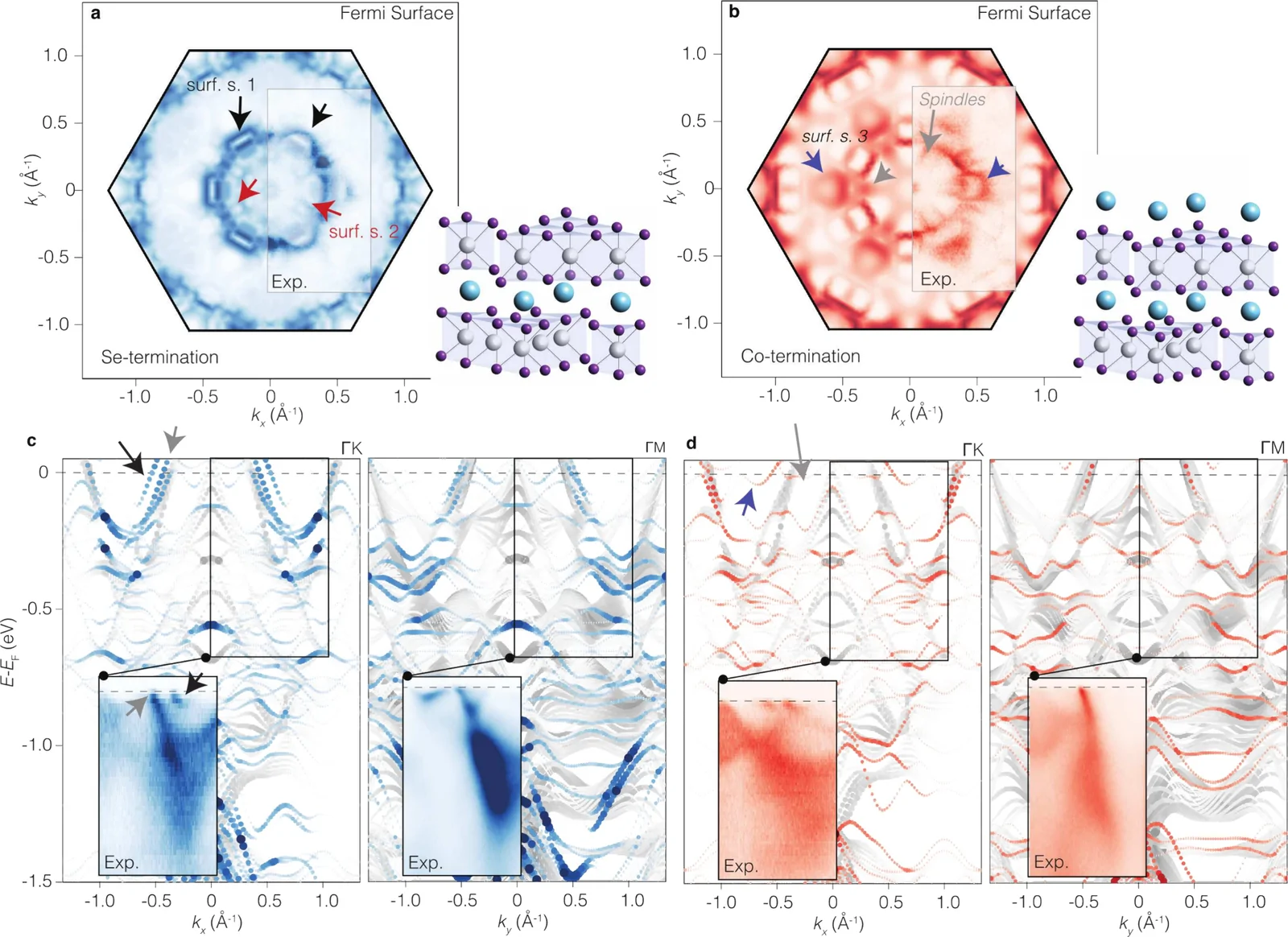
Calculated Fermi surfaces for the (a) Se- and (b) Co-terminated slabs, with the corresponding experimental spectra overlaid in the inset. Both terminations exhibit petal-like pockets (black arrows) and spindle-shaped termination-dependent features (red/green arrows) that form a manifold of surface-derived states and surface resonances, predominantly distributed along the Γ-K directions. No additional bulk-derived bands are found to cross the Fermi level along Γ-K in the same *k*-space region, consistent with the symmetry-constrained band structure. (c) Calculated energy-momentum dispersions for the Se-terminated surface along the Γ-K and Γ-M high-symmetry directions, and (d) the corresponding dispersions for the Co termination. While the petal and spindle features are clearly resolved along Γ-K for both terminations, termination-dependent differences emerge at finite binding energies. Features spectrally separated from the projected bulk continuum are classified as surface-localized states, whereas termination-dependent features overlapping the continuum are described as surface resonances; both categories are consistent with the experimental observations reported in Figure 2.

Note that the experimental electronic structure appears broader and many features visible in the DFT are smeared out. This is likely due to a combination of photoemission matrix elements, a possible contribution from the bulk electronic structure (also shown in Figure 3 in the DFT with the gray shadowing), and presence of local defects and vacancies. Additionally, the broadening is further enhanced by *k*_*z*_ effects, which will necessarily give spectral contribution from a finite portion of the Brillouin zone, as discussed in the Supporting Information. In any case, the strong correspondence between data and calculations provides compelling evidence for the definitive attribution of Co and Se terminations.

To experimentally corroborate the surface state character of the observed features, we measured energy-momentum photoemission spectra along the Γ-M direction for both surface terminations under a range of photon energies and light polarizations. Representative data sets are shown in Figure 4, where the Se-terminated surface exhibits weakly dispersive states just below *E*_*F*_, and the Co-terminated surface shows a deeper, diamond-shaped feature at lower binding energies, fully consistent with the DFT results shown in Figure 3c-d. No additional bands emerge, nor do the observed features display significant changes in dispersion or intensity upon varying either photon energy or polarization. This weak dependence suggests a mixed in-plane and out-of-plane orbital character of the states and, importantly, the absence of any appreciable *k*_*z*_ dispersion, supporting their predominantly two-dimensional, surface-derived or surface-resonance character.

**Figure 4:**
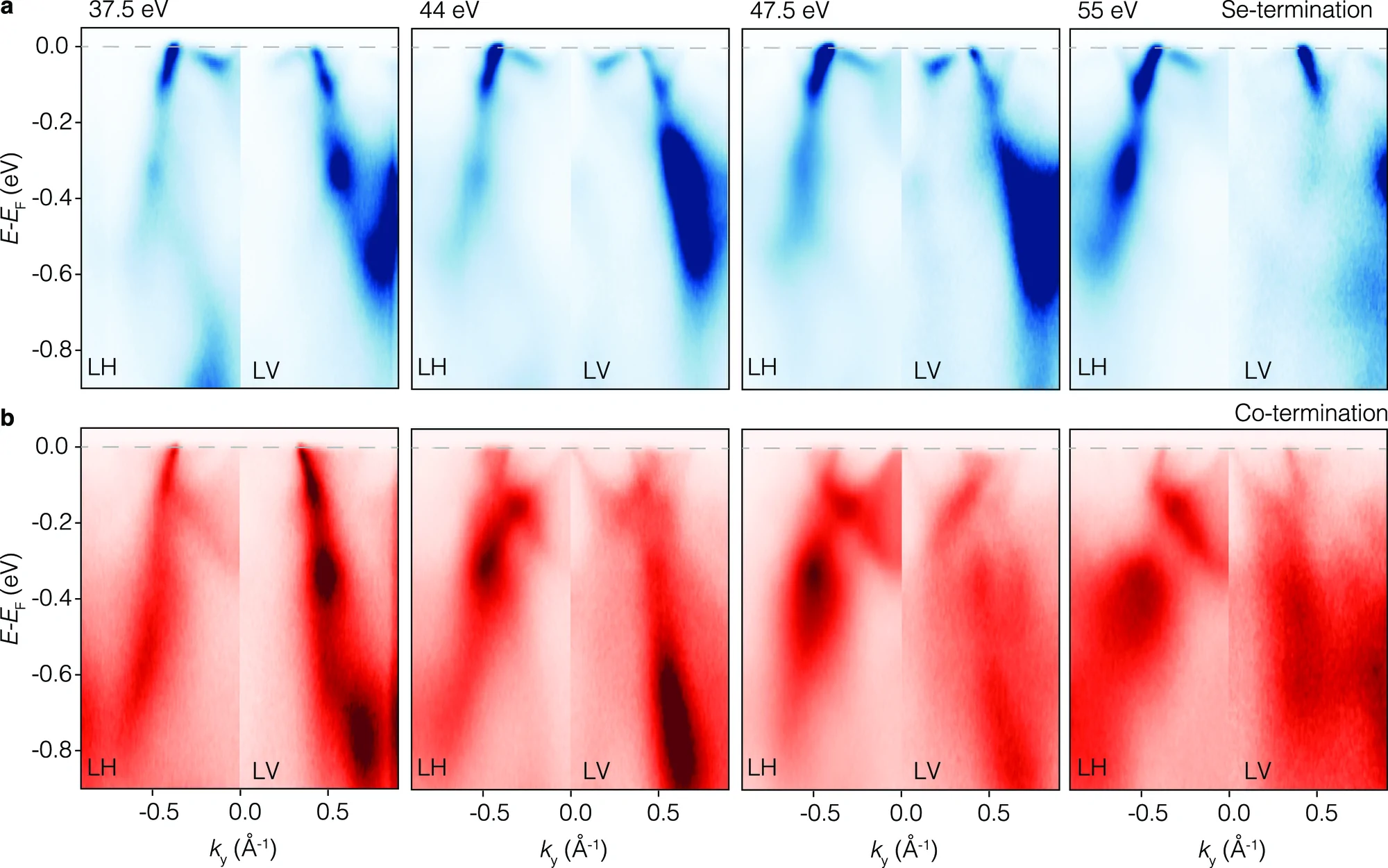
Energy-momentum photoemission spectra along Γ-M as a function of photon energy, for the Se- and Co-terminated surfaces. Measurements are performed with linearly polarized light along the vertical (*s*-polarization) and horizontal (*p*-polarization) directions, to address bands with different symmetries.

In conclusion, we demonstrated that the cleaved surface of CoNb_4_Se_8_ intrinsically hosts two inequivalent terminations, each giving rise to a distinct manifold of surface-derived electronic states and surface resonances. By combining micro-ARPES with micron-scale spatial selectivity and density functional theory calculations, we directly disentangle the electronic structures associated with the Se- and Co-terminated areas and show that their Fermi surfaces, dispersions, and low-energy states differ qualitatively rather than by a simple rigid chemical-potential shift. Termination-specific bands appear depending on the exposed atomic layer, producing petal- and spindle-like features along Γ-K and additional flat or diamond-shaped states along Γ-M that are confirmed by both theory and experiment. The weak photon-energy and polarization dependence establishes the predominantly two-dimensional surface-derived character of these states.

These results highlight that in layered altermagnets, surface contributions are not a secondary effect but a crucial element of the measured photoemission spectra. We note that the presence of surface states does not alter the conclusions regarding the emergence of altermagnetism in this compound.^14,15^ However, without spatially resolved measurements, the coexistence of multiple terminations would lead to spectral averaging and could mimic or obscure the symmetry-driven spin splittings that characterize altermagnetism. Our work therefore provides both a microscopic description of the surface electronic structure of CoNb_4_Se_8_ and a general methodological framework for reliably separating bulk altermagnetic bands from termination-dependent surface-derived states and surface resonances. This approach will be essential for the robust spectroscopic identification of altermagnetism across the growing family of layered compounds and for accurately reconstructing their intrinsic Fermiology.

## Supplementary Material



## Notes

Additional experimental and theoretical details, including the procedure for sample growth, additional XPS-PEEM and LEED measurements on Co- and Se-terminated surfaces, EDS and XPS with large spot size to characterize the overall sample condition, ARPES constant energy maps on the two terminated surfaces to highlight the behavior of the surface states in the *k*-distribution, discussion on the spatial uniformity of terminations in relation to the analysis of micro-ARPES data, and the description of DFT modeling of the slabs, including the choice of supercell parameters (PDF)
