# Supplementary material for: Termination-Dependent Surface Fermiology in the Intercalated Altermagnet CoNb4Se8
Source: Nano Lett. 2026 Oct 19;26(43):14813–20. doi: 10.1021/acs.nanolett.6c01819 (PMC13741913; doi:10.1021/acs.nanolett.6c01819)
Supplement: Supplementary file 1 [file nl6c01819_si_001.pdf]

Supporting Information: Termination-dependent surface Fermiology in the intercalated altermagnet $CoNb_4Se_8$

Pietro Maria Forcella*,[1] Alessandro De Vita*,[2] Matthew D. Watson,[3] Sammar Tayyab,[4] Chiara Bigi,[5] Iulia Cojocariu,[6,7] Marcin Szpytma,[6,8] Matteo Jugovac,[9] Andrea Locatelli,[6] Tevfik Onur Menteş,[6] My Bang Petersen,[10] Paolo Moras,[9] Polina M. Sheverdyaeva,[9] Christopher Candelora,[11] Ilija Zeljkovic,[11] Federico Motti,[12] Jill A. Miwa,[10] Vincent Polewczyk,[13] Matthieu Jamet,[14] Matteo Calandra,[15,*] Younghun Hwang,[16,17,†] Davide Romanin,[1,‡] and Federico Mazzola[18,9,§]

[1]*Université Paris-Saclay, CNRS, Centre de Nanosciences et de Nanotechnologies 91120, Palaiseau, Paris, France*

[2]*Fritz Haber Institut der Max Planck Gesellschaft, Faradayweg 4–6, 14195 Berlin, Germany*

[3]*Diamond Light Source Ltd, Harwell Science and Innovation Campus, Didcot, OX110DE, United Kingdom*

[4]*Dipartimento di Fisica, Sapienza Università di Roma, P.le Aldo Moro 2, 00185 Rome, Italy*

[5]*Synchrotron SOLEIL, L'Orme des Merisiers, Départementale 128, F-91190 Saint-Aubin, France*

[6]*Elettra - Sincrotrone Trieste S.C.p.A., Strada Statale 14, km 163.5, 34149 Trieste, Italy*

[7]*Dipartimento di Fisica, Università degli Studi di Trieste, 34127, Trieste, Italy*

[8]*Faculty of Physics and Applied Computer Science, AGH University of Krakow, Władysława Reymonta 19, 30-059 Krakow, Poland*

[9]*CNR-Istituto di Struttura della Materia (CNR-ISM), Strada Statale 14, km 163.5, 34149 Trieste, Italy*

[10]*Department of Physics and Astronomy, Interdisciplinary Nanoscience Center, Aarhus University, 8000 Aarhus C, Denmark*

[11]*Department of Physics, Boston College, Chestnut Hill, MA 02467, USA*

[12]*CNR-Istituto Officina dei Materiali (IOM), Unità di Trieste, Strada Statale 14, km 163.5, 34149 Basovizza (TS), Italy*

[13]*Université Paris-Saclay, UVSQ, CNRS, GEMaC, 45 Av. des États Unis, 78000 Versailles, France*

[14]*Univ. Grenoble Alpes, CEA, CNRS, IRIG-SPINTEC,*
17 Av. des Martyrs, 38054 Grenoble, France
[15]*Department of Physics, University of Trento,*
Via Sommarive 14, Povo 38123, Italy
[16]*Center for Artificial Low-Dimensional Electronic Systems,*
Institute for Basic Science (IBS), Pohang 37673, Republic of Korea
[17]*Electricity & Electronics and Semiconductor Engineering,*
Ulsan College, Ulsan 44610, Republic of Korea
[18]*Department of Physics and Astronomy 'Galileo Galilei',*
University of Padova, Via Francesco Marzolo, 8, 35121 Padova, Italy

A. Growth details

High-purity Co (99.99%), Nb (99.999%), and Se (99.9999%) powders were used as starting materials. Prior to loading, the quartz ampoule was chemically cleaned and vacuum-annealed to minimise contamination and residual oxygen. The elements were sealed in an evacuated quartz ampoule (10,mm diameter, 150,mm length) together with iodine (5,mg/cm^3 relative to ampoule volume) serving as the transport agent. Crystal growth was carried out in a two-zone horizontal furnace, with the source zone kept at a higher temperature than the growth zone. For $CoNb_4Se_8$, the source region was maintained at 960–980,°C, while the growth region temperature was gradually increased from 880,°C to 900,°C over 100 hours. Both zones were then held at constant temperature for an additional 300 hours to promote the formation of large single crystals. The furnace was subsequently cooled over 100 hours to 200,°C (source) and 100,°C (growth), after which the ampoule was allowed to return to room temperature naturally. Typical crystals obtained under these conditions measured approximately 5,$\times$,5,$\times$,0.1,mm^3. Residual iodine was removed by rinsing the crystals in methanol. The Co concentration x in Co_xNbSe_2 was first estimated using energy-dispersive x-ray spectroscopy (EDS) in a field-emission scanning electron microscope. This methodology is the same shown in Ref.[1]. Samples' compositions were monitored by using XPS, as shown in the main text, and EDS from scanning electron microscope. Their magnetic properties where also determined by SQUID as shown in our previous work in Ref.[1].

B. Core level analysis and terminations

We analyse the change in the XPS intensity ratio between Se and Co core levels measured by micro-ARPES and reported in Fig.1 of the main text. The core level spectra were fitted using Lorentzian line shapes, together with a linear background, in order to extract the integrated peak intensities. This yields

$$R_0 = \left(\frac{I_{\text{Se}}}{I_{\text{Co}}}\right)_0 = 2.41589, \qquad R = \left(\frac{I_{\text{Se}}}{I_{\text{Co}}}\right) = 1.79734. \tag{1}$$

* m.calandrabuonaura@unitn.it
† younghh@uc.ac.kr
‡ davide.romanin@universite-paris-saclay.fr
§ federico.mazzola@unipd.it

Assuming that the screening of the Se signal due to the overlying Co in the Co-terminated surface is negligible, the variation of the ratio reflects a change in the Co photoemission intensity. The relative increase of the Co signal is therefore

$$\frac{I_{\mathrm{Co}}}{I_{\mathrm{Co},0}} = \frac{R_0}{R} = \frac{2.41589}{1.79734} = 1.34415, \tag{2}$$

which corresponds to

$$\frac{\Delta I_{\mathrm{Co}}}{I_{\mathrm{Co},0}} = 0.34415, \tag{3}$$

i.e. a 34.4% enhancement of the Co signal.

To relate this increase to a surface Co coverage, we employ a standard exponential attenuation model. The photoemission intensity from a periodic stack of equivalent Co-containing planes at depths nd is

$$I_{\mathrm{Co},0} \propto \sum_{n=1}^{\infty} e^{-nd/\Lambda} = \frac{e^{-d/\Lambda}}{1 - e^{-d/\Lambda}}, \tag{4}$$

where d is the spacing between successive Co planes along the surface normal, and

$$\Lambda = \lambda \cos\theta \tag{5}$$

is the effective attenuation length, with λ the inelastic mean free path and θ the emission angle.

We then introduce an additional Co contribution located in the outermost surface layer. The surface coverage is denoted by Θ, expressed in monolayers, where one fully occupied allowed Co surface layer corresponds to 0.25 ML. The corresponding intensity contribution is

$$I_{\mathrm{surf}} \propto \frac{\Theta}{0.25}, \tag{6}$$

since it is not attenuated by overlying material.

The total Co signal becomes

$$I_{\mathrm{Co}} = I_{\mathrm{Co},0} + I_{\mathrm{surf}}, \tag{7}$$

and the ratio between the modified and reference intensities is

$$\frac{I_{\mathrm{Co}}}{I_{\mathrm{Co},0}} = 1 + \frac{I_{\mathrm{surf}}}{I_{\mathrm{Co},0}} = 1 + \frac{\Theta/0.25}{e^{-d/\Lambda}/(1 - e^{-d/\Lambda})}. \tag{8}$$

This can be rewritten as

$$\frac{I_{\mathrm{Co}}}{I_{\mathrm{Co},0}} = 1 + \frac{\Theta}{0.25}\left(e^{d/\Lambda} - 1\right). \tag{9}$$

Using the experimental value $I_{\mathrm{Co}}/I_{\mathrm{Co},0} = 1.34415$, we obtain

$$1+\frac{\Theta}{0.25}\left(e^{d/\Lambda}-1\right)=1.34415, \tag{10}$$

which yields

$$\Theta = 0.25\,\frac{0.34415}{e^{d/\Lambda}-1}. \tag{11}$$

For typical XPS conditions, the first Co plane lies at a small fraction of an attenuation length below the surface, such that

$$\frac{d}{\Lambda}\approx 0.28\text{–}0.33. \tag{12}$$

Inserting this range gives

$$\Theta \approx 0.22\text{–}0.27\ \mathrm{ML}, \tag{13}$$

with a central value

$$\Theta \approx 0.25\ \mathrm{ML}. \tag{14}$$

The decrease of the XPS Se/Co intensity ratio from 2.41589 to 1.79734 thus corresponds to a 34.4% enhancement of the Co signal and is quantitatively consistent with an almost fully occupied allowed Co surface layer. The result of this analysis is shown in Fig.S1. Note that this procedure can only be considered with a large uncertainty, specifically associated to the mean free path variation and also to the possibility of additional Se peaks tailing off into the Co region, being these very close to each-other. Thus, despite the tentative quantification, the aim of this analysis is to rather give a qualitative trend.

C. Constant energy maps and binding energy comparison

Figure S2 reports a series of constant-energy maps for the Se- and Co-terminated surfaces, acquired under identical experimental conditions. The purpose of this analysis is to test whether the differences observed in the Fermi surfaces of the two terminations can be reconciled by a simple rigid chemical-potential shift. For the Se-terminated surface (Fig. S2a), the Fermi surface at $E - E_F = 0\,\mathrm{meV}$ exhibits a flower-like contour with pronounced petal-shaped segments. Upon moving to $E - E_F = -25\,\mathrm{meV}$, the central pocket increases in size, consistently with a hole-like dispersion. For the Co-terminated surface (Fig. S2b), analogous constant-energy maps are shown at $E - E_F = -40\,\mathrm{meV}$, $-50\,\mathrm{meV}$, and $-60\,\mathrm{meV}$. As discussed in the main text, the Co termination exhibits an overall shift of approximately $40\,\mathrm{meV}$ relative to the Se termination.

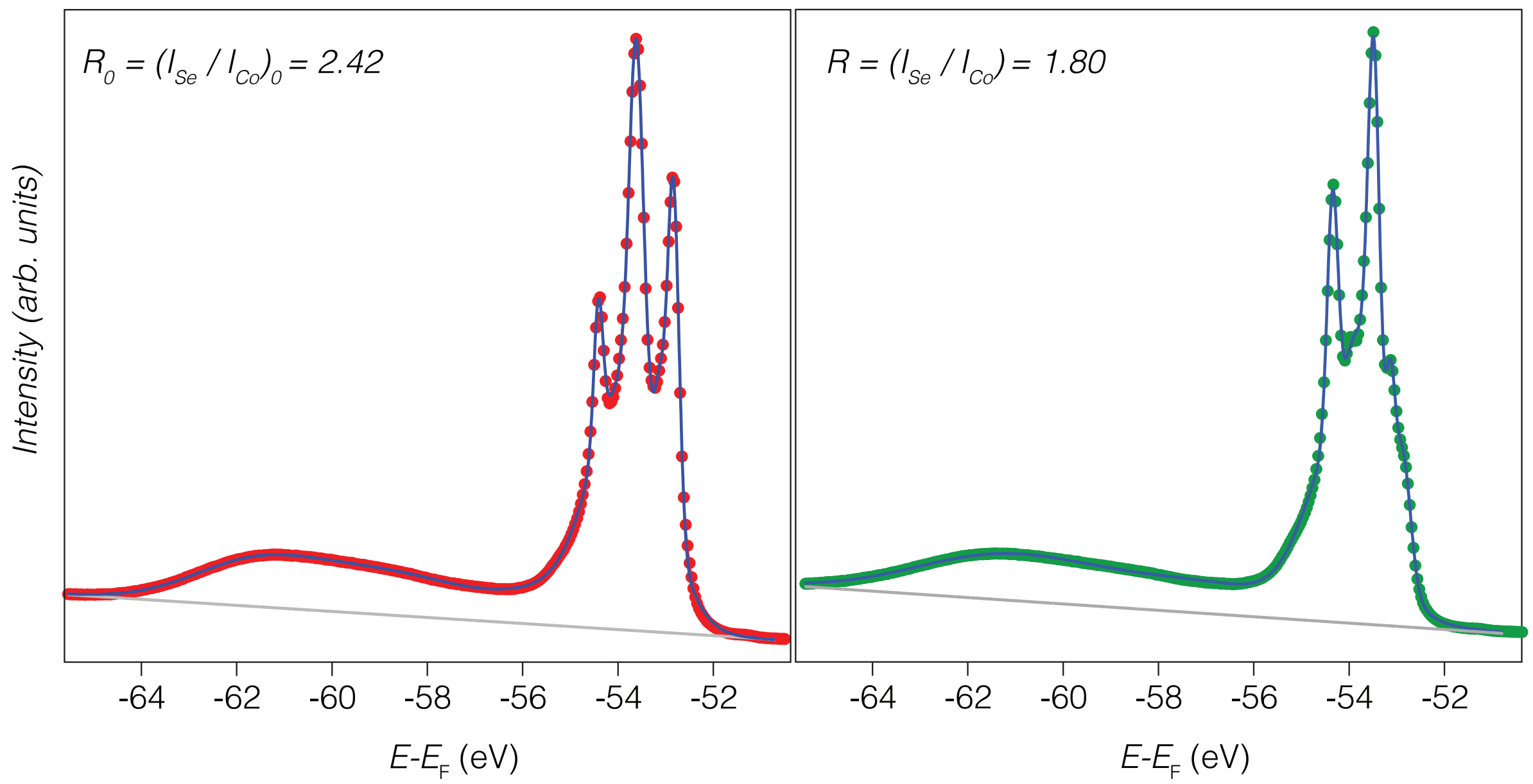

FIG. S1. XPS core-level spectra used to extract the Se/Co intensity ratios. Experimental data (symbols) are fitted with Lorentzian line shapes, including a linear background (grey line), with the total fit shown as solid lines. Left: reference spectrum yielding $R_0 = (I_{\mathrm{Se}}/I_{\mathrm{Co}})_0 = 2.42$. Right: modified surface spectrum yielding $R = (I_{\mathrm{Se}}/I_{\mathrm{Co}}) = 1.80$. The reduction of the Se/Co ratio reflects an enhanced Co contribution at the surface.

For completeness, Fig. S2c compares the Se-terminated map at $E - E_F = -25\,\mathrm{meV}$ with Co-terminated maps at $E - E_F = -65\,\mathrm{meV}$, $-75\,\mathrm{meV}$, and $-85\,\mathrm{meV}$ meV, corresponding to an additional $\sim -60\,\mathrm{meV}$ downward shift.

Under a purely rigid energy displacement, matching the area of the central pocket should simultaneously align the remaining dispersive features, preserving their number and connectivity. While the central pocket size can indeed be approximately matched, the outer petal-like structures and associated intensity segments do not superimpose between the two terminations. Their shape, symmetry distribution, and relative spectral weight remain qualitatively distinct even after compensating for the chemical-potential offset. This analysis unambiguously demonstrates that the termination-dependent Fermi surfaces cannot be obtained from one another by a uniform shift in E_F. Instead, the two terminations host intrinsically different surface electronic structures, consistent with the slab DFT calculations presented in Fig. 3 of the main text.

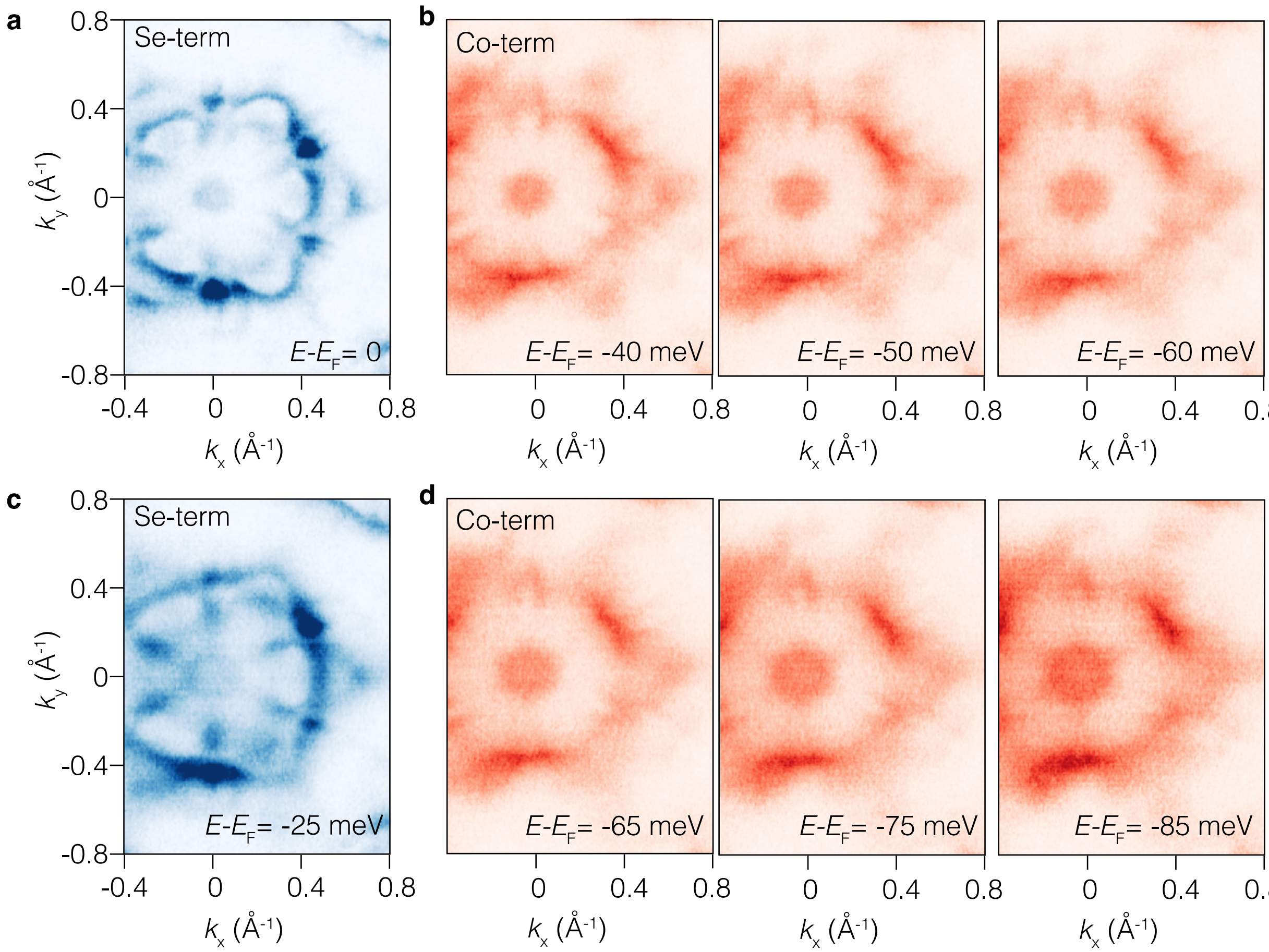

FIG. S2. Constant-energy maps acquired by micro-ARPES for the two surface terminations. **a.** Se-terminated surface at $E - E_F = 0\,\mathrm{eV}$. **b.** Co-terminated surface at $E - E_F = -40\,\mathrm{meV}$, $-50\,\mathrm{meV}$, and $-60\,\mathrm{meV}$. **c.** Se-terminated surface at $E - E_F = -25\,\mathrm{meV}$. **d.** Co-terminated surface at $E - E_F = -65\,\mathrm{meV}$, $-75\,\mathrm{meV}$, and $-85\,\mathrm{meV}$ meV. The first binding-energy offset between the two terminations is chosen to approximately compensate the $\sim 40\,\mathrm{meV}$ chemical-potential shift discussed in the main text, and the other panels want to provide an additional comparison. While the central pocket area can be matched under this shift, the remaining features do not overlap, demonstrating that the distinct Fermiologies of the two terminations cannot be explained by a rigid energy displacement.

D. Broadening effects in real experiment

In angle-resolved photoemission, the out-of-plane momentum k_z is not a good quantum number of the photoelectron final state due to the finite escape depth λ. As a consequence, the experiment does not probe a single k_z, but rather a distribution centred at k_{z0} with a finite width

$$\Delta k_z \sim \frac{1}{\lambda}. \tag{15}$$

The measured photocurrent at fixed in-plane momentum $k_\parallel$ is therefore effectively a weighted average over a range of k_z,

$$I(k_\parallel, \omega) \propto \int dk_z \, W(k_z - k_{z0}) \, A(k_\parallel, k_z, \omega), \tag{16}$$

where $A(k_\parallel, k_z, \omega)$ is the spectral function and W is a broad function of width Δk_z.

This effect becomes particularly pronounced in materials with a large lattice parameter c, as in the case of $CoNb_4Se_8$ for which the Brillouin zone height is reduced to

$$\frac{2\pi}{c}. \tag{17}$$

In this case, even a moderate Δk_z can represent a substantial fraction of the full k_z Brillouin zone, i.e.

$$\frac{\Delta k_z}{2\pi/c} \not\ll 1, \tag{18}$$

so that the measured signal contains contributions from a wide range of inequivalent k_z values.

For bands with finite out-of-plane dispersion, $E = E(k_\parallel, k_z)$, this averaging leads to an intrinsic spectral broadening of order

$$\Delta E \sim \left| \frac{\partial E}{\partial k_z} \right| \Delta k_z, \tag{19}$$

and, more generally, to a redistribution of spectral weight reflecting the underlying k_z-dependent dispersion. In the limiting case where Δk_z spans a significant fraction of the Brillouin zone, the observed spectrum approaches a k_z-integrated signal rather than a well-defined k_z cut, as reflected in our experimental data.

E. Other possible sources of broadening

The finite linewidth observed in the ARPES spectra can arise from several intrinsic and extrinsic mechanisms. From a many-body perspective, electron-electron interactions lead to a finite quasi-particle lifetime, resulting in an energy-dependent broadening of the spectral function. In addition, electron-phonon coupling provides an additional scattering channel, which can further renormalise the dispersion and contribute to the linewidth, particularly at finite temperature. Scattering from residual impurities or disorder acts as an approximately energy-independent contribution, leading to a nearly constant background broadening of the bands.

Beyond these intrinsic effects, extrinsic factors related to the surface quality are expected to play a significant role. In particular, for a Co-terminated surface of $CoNb_4Se_8$, the cleaving process may preferentially remove or displace Co atoms, which are comparatively less strongly bound. This can result in a finite density of defects and vacancies at the surface, giving rise to additional scattering channels. Such disorder-induced scattering leads to a predominantly homogeneous broadening of the spectral features. Furthermore, deviations from the ideal surface stoichiometry, such as Co deficiency, may locally modify the electronic potential and charge balance, thereby slightly shifting the chemical potential. As a consequence, the exact position of the Fermi level with respect to the calculated electronic structure may also be affected.

F. DFT details

Density Functional Theory (DFT) calculations were carried out within the collinear spin–polarized framework using the plane–wave pseudopotential approach as implemented in the QUANTUM ESPRESSO package [2–4]. The electron–ion interaction was described using a norm–conserving pseudopotential for Se atoms, while ultrasoft pseudopotentials were adopted for Nb and Co atoms. The Perdew–Burke–Ernzerhof (PBE) generalized gradient approximation was used for the exchange–correlation functional [5]. A kinetic–energy cutoff of 45 Ry for the wavefunctions and 540 Ry for the charge density was employed.

Integration over the Brillouin zone of the bulk Co–doped 2×2 $NbSe_2$ supercell in the altermagnetic phase was performed using a $9 \times 9 \times 16$ Monkhorst–Pack k–point grid together with a first–order Methfessel–Paxton smearing of 0.005 Ry [6]. The resulting electronic dispersion was subsequently unfolded into the primitive 1×1 $NbSe_2$ Brillouin zone through the unfolding procedure implemented in the `unfold.x` code [7, 8]. The spectral weight, defined as the projection of the supercell eigenstates onto the Bloch states of the primitive cell at fixed $\mathbf{k}$, is used to construct the unfolded electronic dispersion. The continuous unfolded spectral-intensity maps and calculated Fermi-surface images were generated on an energy grid with step $\Delta E = 0.005$ eV by replacing each eigenvalue E_n with a Lorentzian spectral function $L(E-E_n) = \pi^{-1}w[(E-E_n)^2+w^2]^{-1}$, using a broadening parameter $w = 0.005$ eV. The unfolding was performed from the $2 \times 2 \times 1$ supercell to the primitive Brillouin zone. To evaluate surface effects with different terminations we constructed slab geometries. For the Se termination we employed a slab consisting of five $NbSe_2$ layers with four Co atoms and a vacuum region of 45 Å along the non–periodic direction

perpendicular to the surface. For the Co termination we used a slab composed of three $NbSe_2$ layers with four Co atoms and a vacuum region of 55 Å. The slab thickness was chosen in order to preserve the altermagnetic configuration while simultaneously allowing the realization of both surface terminations. It is worth noting that, because of the unfolding into the primitive zone, which is hexagonal, the calculated electronic structure also displays a hexagonal symmetry pattern. At the surface, however, a real experiment would capture the effect of symmetry breaking, resulting in a three-fold symmetry, as observed in the constant-energy maps measured by micro-ARPES.

For the slab calculations the atomic positions were fully relaxed by increasing the kinetic–energy cutoff to 60 Ry for the wavefunctions and 720 Ry for the charge density. Structural optimization was performed until the residual forces on each atom were smaller than 10^{-4} Ry/Bohr. The ground–state electronic density was obtained using a $9 \times 9 \times 1$ k–point mesh with a first–order Methfessel–Paxton smearing of 0.005 Ry. Atom-projected DOS curves were obtained from the relaxed slab calculations by projecting the Kohn–Sham states onto Nb, Se, and Co atomic contributions for each termination. To assess the surface-versus-bulk character of the low-energy spectral weight, we also compared projected DOS from the outer surface layers with DOS from inner, bulk-like layers of the slab.

G. DFT bulk versus surface states

To disentangle the origin of the electronic states observed experimentally, we compare bulk and slab DFT calculations, explicitly accounting for the two possible surface terminations.

The bulk electronic structure is characterised by dispersive bands with finite k_z dependence, reflecting the three-dimensional periodicity of the crystal. When projected onto the surface Brillouin zone, these states form a continuum of spectral weight. This bulk continuum is used as the reference against which the slab bands are classified.

The slab calculations reveal termination-dependent additional spectral weight. We classify a slab feature as an isolated surface-localised state only when it appears in an energy–momentum region where no bulk band is present in the bulk projection and when its dispersion or spectral weight depends on the termination. If a termination-dependent feature overlaps the projected bulk continuum, we instead refer to it as a surface resonance. This distinction avoids assigning a strictly surface-localised character to features, such as the central $\bar{\Gamma}$-centred pocket, whose spectral weight can overlap the bulk projection.

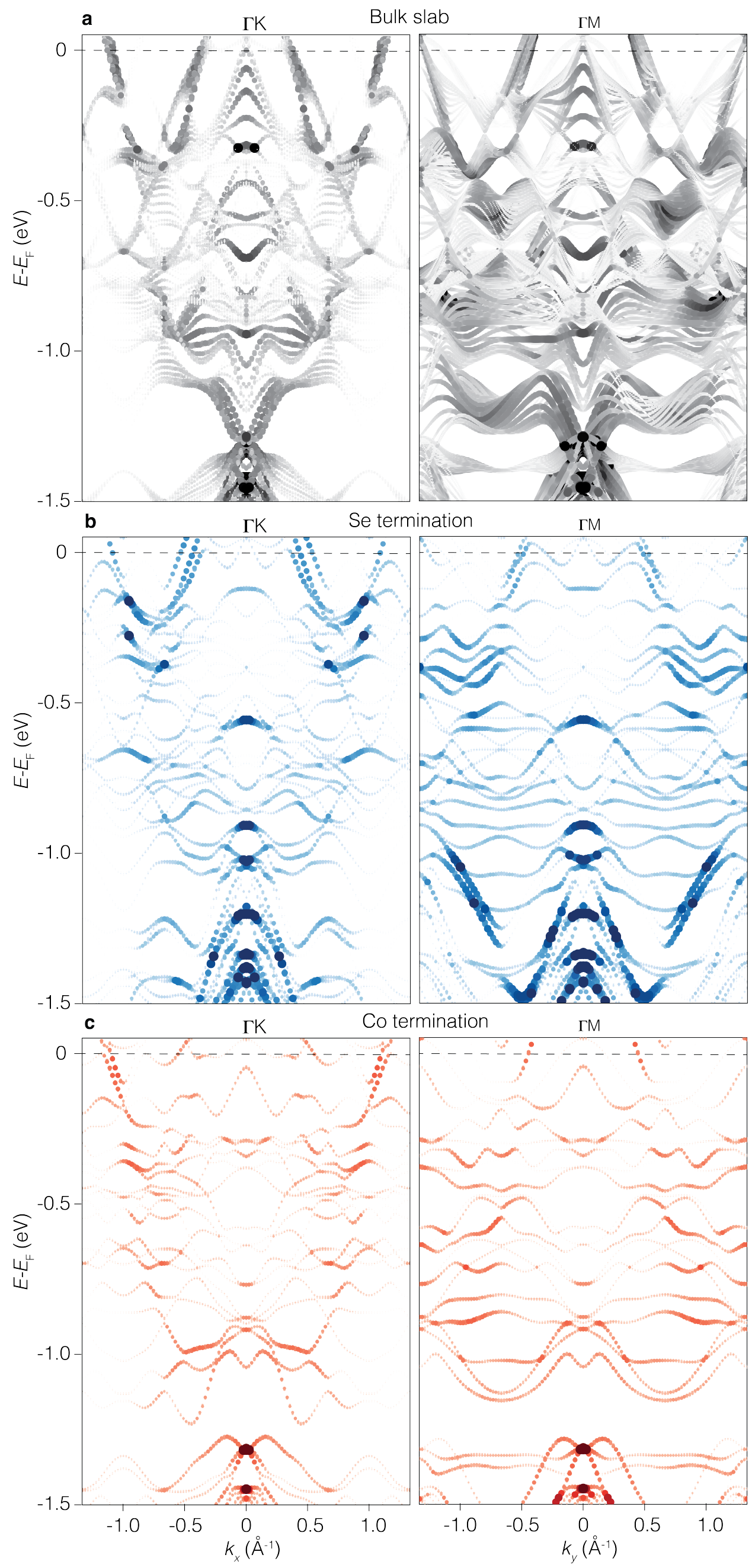

FIG. S3. DFT electronic structure comparison between bulk and slab geometries. (a) Bulk band structure projected along the $\bar{\Gamma}-\bar{K}$ and $\bar{\Gamma}-\bar{M}$ directions, showing the continuum of bulk states. (b) Slab calculation for the Se-terminated surface, where additional weakly dispersive states appear below E_F, particularly along $\bar{\Gamma}-\bar{M}$. (c) Slab calculation for the Co-terminated surface, exhibiting a distinct set of termination-dependent bands shifted to higher binding energy and with modified dispersion. The comparison distinguishes features spectrally separated from the projected bulk continuum, which are classified as surface-localised states, from termination-dependent features overlapping the continuum, which are described as surface resonances.

For the Se-terminated slab, the calculations show weakly dispersive bands located just below E_F, particularly evident along the $\bar{\Gamma} - \bar{M}$ direction. These states form relatively flat features that are termination dependent but partly embedded within the projected bulk continuum, and are therefore best described as surface resonances in the overlapping regions. For the Co-terminated slab, the corresponding states are shifted to higher binding energy and acquire a distinct diamond-like dispersion, indicating a different surface potential and hybridisation.

Along the $\bar{\Gamma} - \bar{K}$ direction, both terminations exhibit surface-derived bands that approach and cross the Fermi level, forming the characteristic petal- and spindle-like features observed in the calculated Fermi surfaces. Where these features are spectrally separated from the projected bulk continuum, the slab calculations support their identification as surface-localised states.

A direct comparison between bulk and slab calculations shows that the termination-dependent features cannot be obtained from the bulk band structure by a rigid energy shift or simple projection. Instead, they arise from the modified surface potential and from surface–bulk hybridisation, with their detailed shape and energy position controlled by the termination.

These results demonstrate that the low-energy electronic structure near the Fermi level contains a significant contribution from termination-dependent surface-derived states and surface resonances. The central $\bar{\Gamma}$-centred pocket is used in the main text only as a registration feature for the rigid-shift analysis and is not used as evidence for the isolated surface-state assignment of the other bands.

H. Spatial uniformity of spectroscopic signals

To further expand from Fig. 1 of the main text the discussion on the spatial variation of surface terminations, we have plotted micro-ARPES spectra acquired at several different positions of the spatial map, as shown in Figure S4 below.

The spatial maps in Fig. 1b of the main text and in Fig. S4 are constructed by raster-scanning the micro-ARPES beam across the sample. At each spatial coordinate (x, y), the local fingerprint intensity is obtained by integrating the measured photoemission intensity over a fixed energy–momentum window,

$$I_{\text{fp}}(x, y) = \iint_W I(x, y; k, E)\, dk\, dE, \qquad W = \{E_1 \leq E \leq E_2,\ k_1 \leq k \leq k_2\}. \tag{20}$$

The window is selected to isolate a termination-specific valence-band fingerprint identified

from the slab-DFT calculations and from representative single-domain spectra, rather than a generic chemical contrast. For the map shown here, an example valence-band window is $E \in [43.0, 43.13]$ eV and $k \in [-1.85, -0.85]$ Å^{-1}. A complementary core-level window in the Se $3d$/Co $3p$ region was used as a chemical check, but not as the sole criterion for assignment, because core-level maps can include bulk contributions and can retain intensity from tilted or poorly ordered regions. We did not apply an automated intensity-ratio threshold to assign the spots. Instead, the map was used as a practical spatial guide to locate candidate domains, and each selected position was accepted only when the manually focused micro-ARPES spectrum showed both the appropriate core-level lineshape and the sharp near-E_F termination fingerprint. Tilted or disordered regions were rejected because they lacked the well-centred, coherent dispersion of a single termination.

Positions 1 and 2, in the homogeneous brown–yellow region, show clean, coherent dispersive bands — the well-ordered single-termination spectra. Each displayed spectrum is a single micro-ARPES acquisition from the circled position and is not itself an integrated spatial map. For the high-resolution measurements the beamline conditions were optimized spot-by-spot, including analyser focus, beam focus, slits, and acquisition conditions, and the data were acquired in swept mode to suppress detector artefacts. No additional algorithmic treatment was used for the termination assignment. Position 3 looks very similar but with a slight distortion we attribute to a possible tilt of the flake and a slightly larger disorder, as corroborated by an increased broadening of the spectra. Position 4 shows the same onset of tilt together with noticeably increased broadening, consistent with approaching a less well-ordered area. Position 5, in the dark-blue corner at the edge, is essentially featureless noise — labelled "out," *i.e.*, the beam is out of the sample. Positions 6 and 7, in the bright-white region, show a clearly different dispersion ("different surface") — the other termination.

Figure S4 makes the order/disorder-plus-termination picture explicit and visual: a central well-ordered single-termination domain (1, 2) giving sharp coherent bands; a gradient toward the edges where flake tilting and the onset of disorder progressively broaden and distort the spectra (3, 4); a point off the sample with no signal (5); and a spatially distinct second termination (6, 7) with its own dispersion. This directly supports that coherent spectra come from ordered single-termination patches, whereas by approaching the edges the signal degrades through tilt and disorder rather than through a smooth sub-stoichiometric Co gradient.

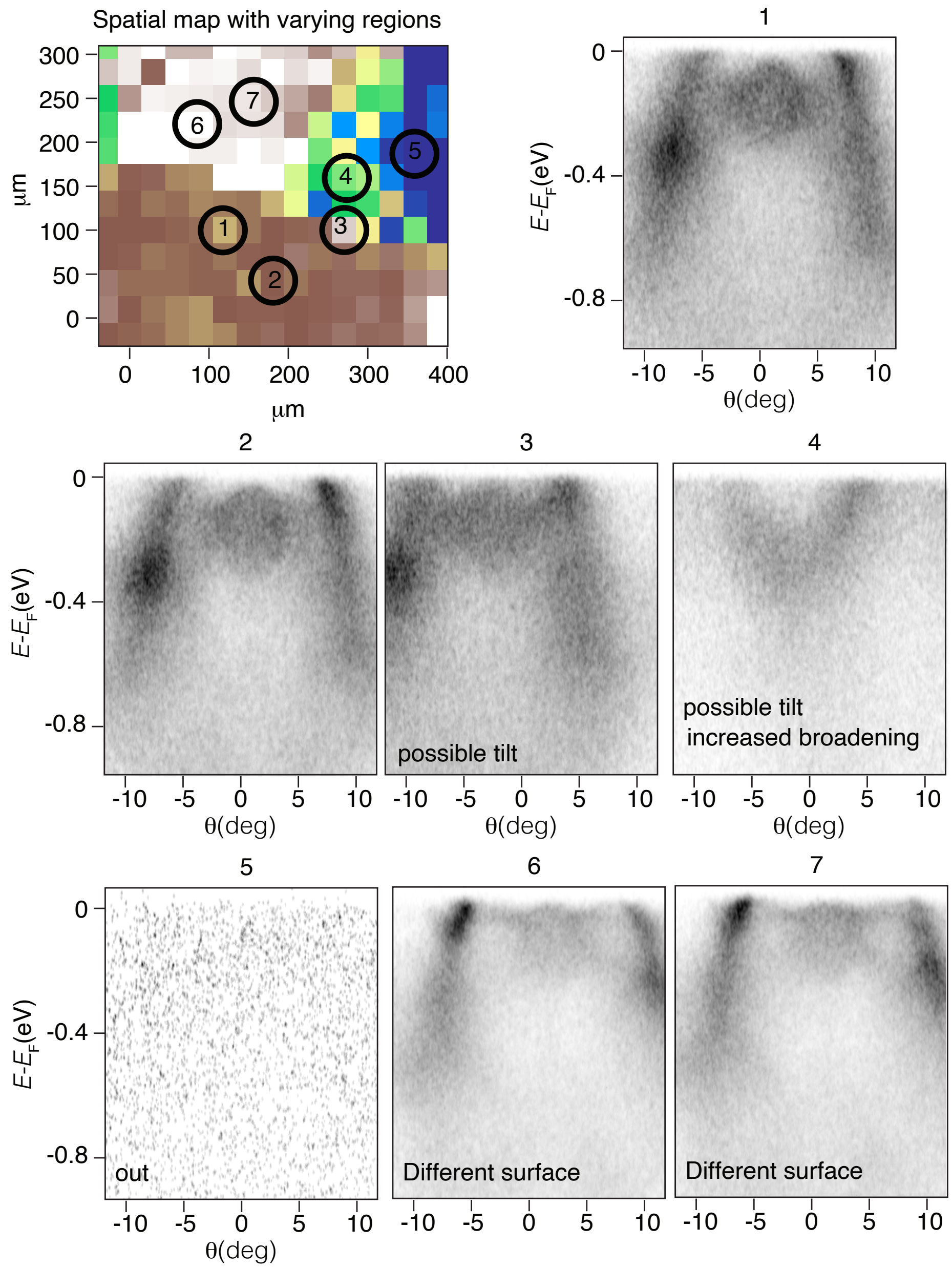

FIG. S4. Spatially-resolved ARPES investigation. The top-left panel is a spatial map ($\approx 400\times300\,\mu$m) obtained by integrating the valence-band intensity over a termination-sensitive window, for example $E \in [43.0, 43.13]$ eV and $k \in [-1.85, -0.85]$ Å^{-1}. The colour therefore encodes a spectral fingerprint used to guide the search for single-termination regions, not an automated intensity-threshold classification. Seven positions are circled (1-7), and the other panels show the micro-ARPES (E, k) dispersions taken at each of those points, plotting $E - E_F$ vs emission angle θ over roughly $\pm10°$.

I. Atom- and layer-projected density of states

We added atom-projected DOS calculations for both slab terminations to clarify the microscopic character of the near-E_F spectral weight. The states close to E_F have mixed atomic character with termination-dependent Nb, Se, and Co contributions. In the Se-terminated slab, the Se-projected component remains sizeable near E_F, whereas in the Co-terminated slab the Co-projected component is enhanced near the Fermi level. We also compare the projected DOS of surface layers with inner, bulk-like layers of the slab. This comparison shows that the near-E_F spectral weight contains both surface-enhanced and bulk-like contributions, supporting the distinction used in the main text between isolated surface-localised states and termination-dependent surface resonances.

[1] A. D. Vita, C. Bigi, D. Romanin, M. D. Watson, V. Polewczyk, M. Zonno, F. Bertran, M. B. Petersen, F. Motti, G. Vinai, M. Tuniz, F. Cilento, M. Cuoco, B. M. Andersen, A. Kreisel, L. J. D'Onofrio, O. J. Clark, M. T. Edmonds, C. Candelora, M. Xu, S. Cheng, A. LaFleur, T. Antonelli, G. Sangiovanni, L. D. Re, I. Vobornik, J. Fujii, F. M. Granozio, A. Sambri, E. D. Gennaro, J. B. Jacobsen, H. Jacobsen, I. Cojocariu, M. Szpytma, A. Locatelli, T. Mentes, M. Jamet, J.-F. Jacquot, P. Orgiani, R. Ernstorfer, I. Zeljkovic, Y. Hwang, M. Calandra, J. A. Miwa, and F. Mazzola, Robust spin splitting and fermiology in a layered altermagnet (2026), arXiv:2502.20010 [cond-mat.str-el].

[2] P. Giannozzi, S. Baroni, N. Bonini, M. Calandra, R. Car, C. Cavazzoni, D. Ceresoli, G. L. Chiarotti, M. Cococcioni, I. Dabo, A. D. Corso, S. de Gironcoli, S. Fabris, G. Fratesi, R. Gebauer, U. Gerstmann, C. Gougoussis, A. Kokalj, M. Lazzeri, L. Martin-Samos, N. Marzari, F. Mauri, R. Mazzarello, S. Paolini, A. Pasquarello, L. Paulatto, C. Sbraccia, S. Scandolo, G. Sclauzero, A. P. Seitsonen, A. Smogunov, P. Umari, and R. M. Wentzcovitch, Quantum espresso: a modular and open-source software project for quantum simulations of materials, Journal of Physics: Condensed Matter **21**, 395502 (2009).

[3] P. Giannozzi, O. Andreussi, T. Brumme, O. Bunau, M. B. Nardelli, M. Calandra, R. Car, C. Cavazzoni, D. Ceresoli, M. Cococcioni, N. Colonna, I. Carnimeo, A. D. Corso, S. de Gironcoli, P. Delugas, R. A. D. Jr, A. Ferretti, A. Floris, G. Fratesi, G. Fugallo, R. Gebauer, U. Gerstmann, F. Giustino, T. Gorni, J. Jia, M. Kawamura, H.-Y. Ko, A. Kokalj, E. Küçükbenli, M. Lazzeri, M. Marsili, N. Marzari, F. Mauri, N. L. Nguyen, H.-V. Nguyen, A. O. de-la Roza, L. Paulatto, S. Poncé, D. Rocca, R. Sabatini, B. Santra,

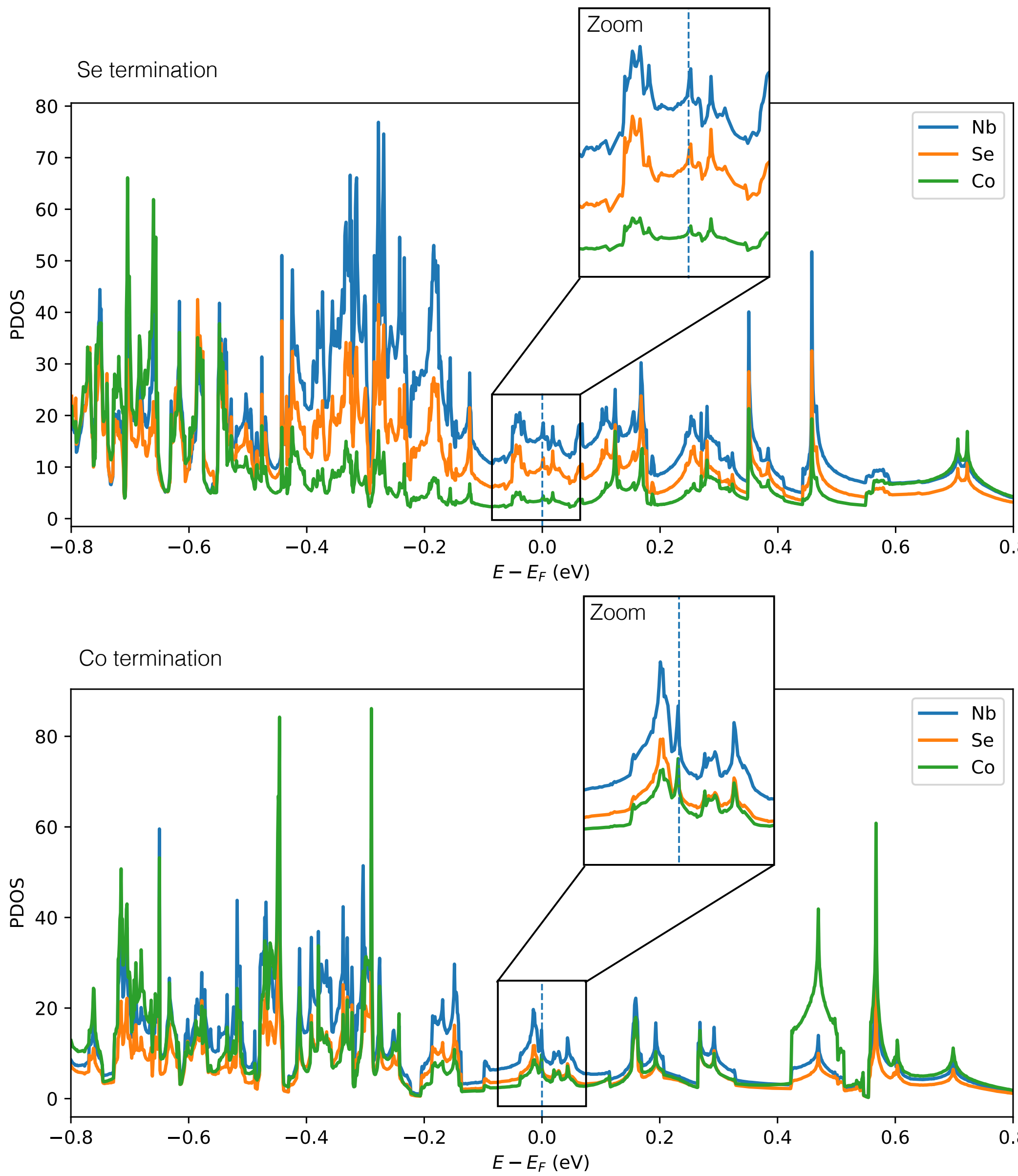

FIG. S5. Atom-projected density of states for the Se-terminated and Co-terminated slab geometries. The PDOS is decomposed into Nb, Se, and Co contributions, with zoomed insets around E_F. The states close to E_F have mixed atomic character, with sizeable Se-projected weight for the Se termination and enhanced Co-projected weight for the Co termination.

M. Schlipf, A. P. Seitsonen, A. Smogunov, I. Timrov, T. Thonhauser, P. Umari, N. Vast, X. Wu, and S. Baroni, Advanced capabilities for materials modelling with QUANTUM ESPRESSO, Journal of Physics: Condensed Matter **29**, 465901 (2017).

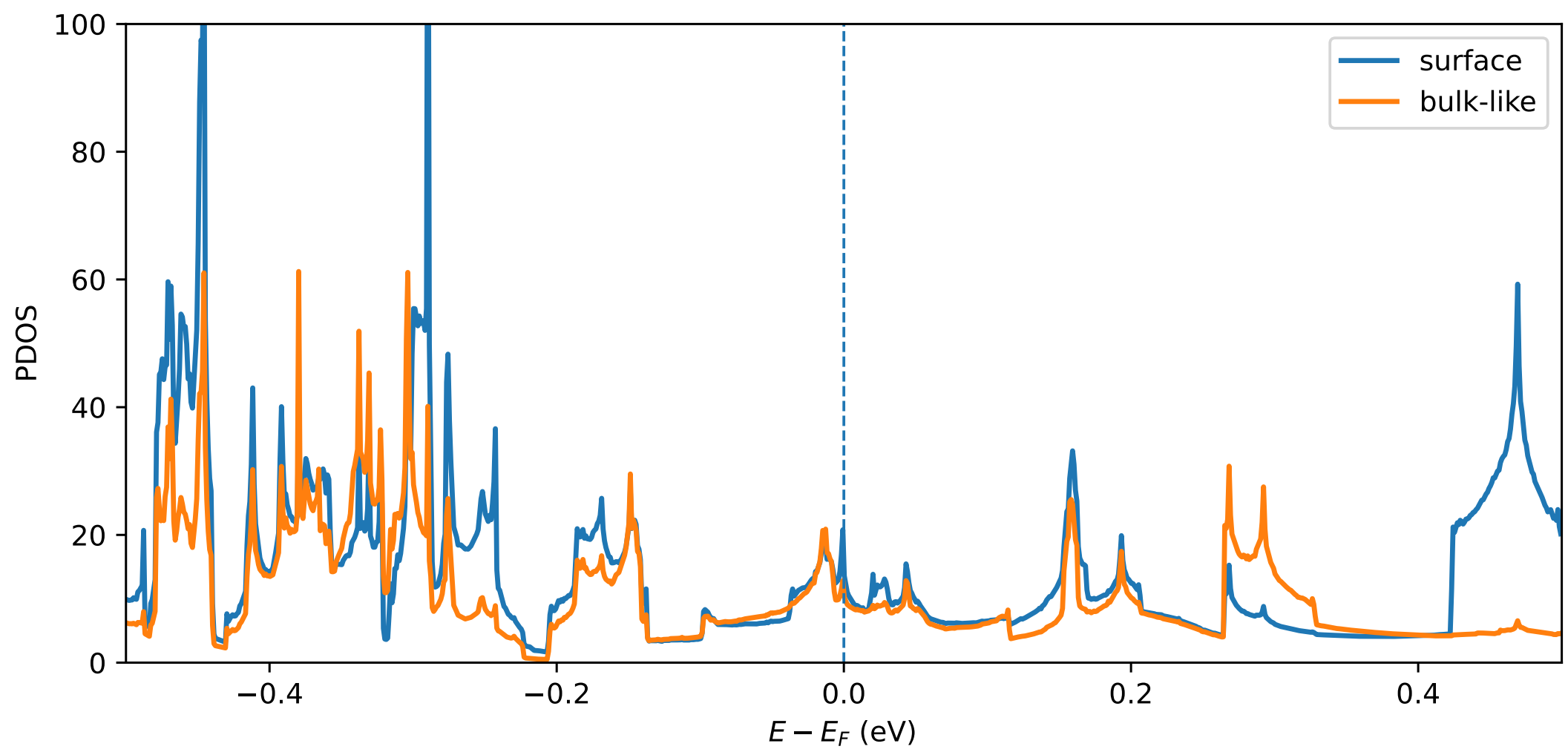

FIG. S6. Surface-layer and bulk-like projected density of states from the slab calculation. The comparison shows that the low-energy spectral weight includes both enhanced surface-layer contributions and bulk-like components near E_F, consistent with describing overlapping termination-dependent features as surface resonances rather than strictly isolated surface states.

[4] P. Giannozzi, O. Baseggio, P. Bonfà, D. Brunato, R. Car, I. Carnimeo, C. Cavazzoni, S. de Gironcoli, P. Delugas, F. F. Ruffino, A. Ferretti, N. Marzari, I. Timrov, A. Urru, and S. Baroni, Quantum espresso toward the exascale, The Journal of Chemical Physics **152**, 154105 (2020).

[5] J. P. Perdew, K. Burke, and M. Ernzerhof, Generalized gradient approximation made simple, Physical Review Letters **77**, 3865–3868 (1996).

[6] M. Methfessel and A. T. Paxton, High-precision sampling for brillouin-zone integration in metals, Physical Review B **40**, 3616–3621 (1989).

[7] V. Popescu and A. Zunger, Extracting E versus $\vec{k}$ effective band structure from supercell calculations on alloys and impurities, Phys. Rev. B **85**, 085201 (2012).

[8] D. Pacilè, C. Cardoso, G. Avvisati, I. Vobornik, C. Mariani, D. A. Leon, P. Bonfà, D. Varsano, A. Ferretti, and M. G. Betti, Narrowing of d bands of feco layers intercalated under graphene, Applied Physics Letters **118**, 10.1063/5.0047266 (2021).
